# Validation of an Analytical Method for the Simultaneous Determination of Hyaluronic Acid Concentration and Molecular Weight by Size-Exclusion Chromatography

**DOI:** 10.3390/molecules26175360

**Published:** 2021-09-03

**Authors:** Luis Antonio Suárez-Hernández, Rosa María Camacho-Ruíz, Enrique Arriola-Guevara, Eduardo Padilla-Camberos, Manuel Reinhart Kirchmayr, Rosa Isela Corona-González, Guadalupe María Guatemala-Morales

**Affiliations:** 1 Tecnología Alimentaria, Centro de Investigación y Asistencia en Tecnología y Diseño del Estado de Jalisco, A.C., Normalistas 800, Guadalajara C.P. 44270, Jalisco, Mexico; lusuarez_al@ciatej.edu.mx; 2 Biotecnología Industrial, Centro de Investigación y Asistencia en Tecnología y Diseño del Estado de Jalisco, A.C., Camino Arenero 1227, Zapopan C.P. 45019, Jalisco, Mexico; mkirchmayr@ciatej.mx; 3 Departamento de Ingeniería Química, CUCEI-Universidad de Guadalajara, Blvd. Marcelino García Barragán 1421, Guadalajara C.P. 44430, Jalisco, Mexico; enrique.arriola@academicos.udg.mx; 4 Biotecnología Médica y Farmacéutica, Centro de Investigación y Asistencia en Tecnología y Diseño del Estado de Jalisco, A.C., Normalistas 800, Guadalajara C.P. 44270, Jalisco, Mexico; epadilla@ciatej.mx

**Keywords:** hyaluronic acid, size-exclusion chromatography, molecular weight distribution, analytical method, *Streptococcus zooepidemicus*

## Abstract

The hyaluronic acid (HA) global market growth can be attributed to its use in medical, cosmetic, and pharmaceutical applications; thus, it is important to have validated, analytical methods to ensure confidence and security of its use (and to save time and resources). In this work, a size-exclusion chromatography method (HPLC-SEC) was validated to determine the concentration and molecular distribution of HA simultaneously. Analytical curves were developed for concentration and molecular weight in the ranges of 100–1000 mg/L and 0.011–2.200 MDa, respectively. The HPLC-SEC method showed repeatability and reproducibility greater than 98% and limits of detection and quantification of 12 and 42 mg/L, respectively, and was successfully applied to the analysis of HA from a bacterial culture, as well as cosmetic, and pharmaceutical products.

## 1. Introduction

Hyaluronic acid (HA) is a linear polysaccharide composed of a repeated disaccharide formed by D-glucuronic acid and N-acetyl glucosamine, linked by β-(1,4) and β-(1,3) bonds [1]. HA is used in the treatment of osteoarthritis, viscosupplementation, ophthalmic surgery, facial and hand rejuvenation, wound healing, tissue engineering, bone regeneration, dermal fillers and implants, drug delivery, etc. [1,2]. HA can be extracted from animal tissues (rooster comb, human umbilical cord or bovine vitreous body) or produced in bacterial cultures [3,4,5]. The concentration and molecular weight (MW) of HA vary according to the type of animal tissue used for extraction or culture conditions used for microbial production. In both cases, a mixture of polymeric chains with different MW (polydisperse) of HA may be present [4,6].

The rheological features of HA are determined by its concentration and average molecular weight, and determine its applications and biological functions and, consequently, its commercial value [6,7]. Thus, it is essential to have accurate analytical methods to determine the concentration and MW in the production process and purification stages [4,6]. The most used methods to estimate HA concentration are based on hydrolysis of the polymer by acid, alkaline, or enzymatic hydrolysis (indirect methods) [8,9,10,11,12,13], which determine the presence of one of its monomers (usually D-glucuronic acid) by photometric methods. However, these methods are susceptible to interference by residual carbohydrates and proteins from the source tissues or microbial processes, and the results are unreliable [14,15]. Quantification of HA oligomers, products of enzymatic hydrolysis by MALDI-TOF MS [16,17] or HPTLC [18], have been proposed to estimate the HA concentration. However, this is a high-cost method that requires special equipment and prior solvent purification steps, which results in partial loss of the HA polymer. The introduction of derivatized products from HA allows to increase the sensitivity of fluorescence or mass spectrometry-related methods [19,20,21]. However, the derivatization products are unstable, their lifetime is short, sometimes not all of the analyte present in a sample is reacted, and it is not suitable for complex samples [22]. On the other hand, there are absolute methods to evaluate the MW of HA, such as multi-angle laser light-scattering (MALS), so called because they do not require a calibration process or reference substances in the determination. MALS is based on measuring light scattering at many angles and is extremely sensitive at measuring absolute MW. It is usually connected to a chromatographic system, and it is essential that it is connected to a concentration detector (refractive index or UV) since it does not quantify HA. Other methods, such as viscosimetry, gel, capillary electrophoresis, molecular exclusion chromatography, etc., are also available to assess the molecular distribution of HA and are recognized as relative methods as they usually require a calibration process [22,23,24,25,26,27,28]. Methods, such as sedimentation, osmometry, and combinations of these exist, but they present discrepancies in results due to impurities, errors in measurements, and poor calibration processes [14], and typically require a large amount of sample. Recently, NMR-DOSY, NIR and Rayleigh scattering resonance techniques have been described to determine the molecular distribution of HA [29,30,31], but the equipment is poorly accessible due its availability and high cost [32].

Size-exclusion chromatographic separation (SEC) separates molecules in solution according to their size, with high performance in the separation of macromolecules. Additionally, high performance liquid chromatography (HPLC) significantly improves the speed and accuracy of determinations [15,33,34]. HPLC-SEC allows to determine the concentration and molecular distribution of HA simultaneously in an open range from a few to thousands of Daltons (Da). This is a reproducible technique that minimizes the drawbacks associated with light scattering, sedimentation equilibrium, non-Newtonian fluid viscosimetry of HA solutions, and requires a small sample volume for analysis [33,35,36]. This method was proposed by Jagannath and Ramachandran [33], who determined simultaneously the concentration and MW of HA in bacterial culture by HPLC-SEC with an Ultrahydrogel 2000 column at 30°C. However, under these conditions, an exponential fit of the experimental data was necessary for high-MW samples and may result in inaccurate results. In addition, this method does not allow to calculate the polydispersity of HA, and the procedure for estimating HA concentration needs to be detailed. To our knowledge, there are no reports on the validation of an HPLC-SEC analytical method for the simultaneous determination of HA concentration and molecular weight. Based on the above, in this study, the validation of the HPLC-SEC analytical method for the simultaneous evaluation of HA concentration and molecular distribution was carried out. Validated analytical methods provide confidence and certainty in the results, are part of good analytical practices, are a requirement of regulatory agencies and pharmacopeias, and save time and resources [34].

## 2. Results and Discussion

### 2.1. Effect of Column Temperature on HA Resolution in HPLC-SEC Method

Accuracy of the HPLC-SEC method essentially depends on the temperature and the mobile phase (flow and composition) used to process the sample [15,35,36,37,38]. In order to determine the best conditions to quantify the hyaluronic acid (HA), we tested the effect of four column temperatures at 30, 50, 60, and 70 °C on the repeatability and reproducibility of the method using an Ultrahydrogel 2000 column. Samples from culture broth containing HA were evaluated according to USP [34]. A linear dependence between HA concentration and the analytical response of the method (peak area) was found for all temperatures analyzed (correlation coefficient R > 0.99), even though the highest correlation was found at 70 °C (R = 0.9963). With increasing temperature, repeatability and reproducibility increased (Table 1), which are key parameters for international acceptance of analytical methods. It has been shown that by increasing other column temperatures, there is a significant improvement in HA analysis (Shodex OH-Pak SB805-HQ, Shodex SUGAR KS-805) [39,40,41,42].

At elevated temperatures (>60°C) the viscosity of the solutions (mobile phase and sample) is reduced and, consequently, the pressure in the column decreases, the number and resolution of theoretical plates increases, and the adsorption of sample components is reduced [43]. Higher temperature substantially increases the rate of analyte separation due to increased solute diffusion coefficients (mass transfer). This represents a lower load to the pump supplying the mobile phase, allows using a higher flow rate and decreases the analysis time [44]. Jagannath and Ramachandran [33] tested concentrations between 1.9 and 2.9 g/L of HA, but did not detail the process by which these concentrations were estimated (analytical curve and type of data fitting), the previous stages of purification or preparation of the samples for analysis. Therefore, for the following stages, the column temperature was set at 70°C for the analysis of samples with HA.

### 2.2. Validation of the HPLC-SEC Method

The validation of HPCL-SEC method demonstrated that it is sufficiently reliable if the results are obtained under the established conditions. Validation is based on the determination of parameters, such as linearity (correlation coefficient, slope, and intercept), precision, and accuracy expressed by repeatability and reproducibility, and is essential in the analytical practice of regulatory agencies and international pharmacopeias [34]. In this study, HA analytical curves were performed in the range of 100 to 1000 mg/L and linearity was evaluated by calculating the correlation coefficient, slope and intercept, and precision expressed as repeatability and reproducibility (Table 2). Multiple determination coefficients (R^2^) > 0.9992 and adjusted R^2^ > 0.9991 for the analytical curves indicate a high linear dependence between HA concentration and RI detector response in the analyzed concentration range regardless of MW (Pearson correlation coefficient, PCC > 0.9996). Analytical curves were used to estimate the HA concentration in the samples because this method allows to determine the concentration of HA in samples with high accuracy.

The precision of the HPLC-SEC method was evaluated by calculating repeatability and reproducibility. The method showed a repeatability 98.3% on average, a coefficient of variation (CV) between 0.8–1.7% and reproducibility greater than 98% for all HA standards in the range of concentrations analyzed. These results demonstrate that the method complies with the international acceptance criteria that establish a CV < 5%, which means that the method allows identifying and quantifying HA reliably.

The instrumental limits of detection (LOD) and quantification (LOQ) were calculated based on the signal-to-noise ratio of the method (Table 2). The increase in the MW of HA increased the LOD and LOQ values. It means that the method has higher analytical sensitivity and accuracy to detect and quantify HA of higher MW and it has been reported to be a phenomenon related to the randomness of the RI detector response with randomness due to concentration variation of high MW or highly polydisperse samples [45]. The sensitivity of any relative analytical method (requiring calibration) is related to the methodology used for the analytical curve. It is known that the higher the slope, the higher the sensitivity of the method, while the value of the intercept is directly related to the presence of interferences or systematic errors and must include zero to comply with the proportionality requirement. The higher the absolute value of the intercept, the greater the error produced by interferences in the analytical method [34,45].

It is also important to consider that HA concentration and MW can lead to inadequate fractionation of molecules due to intermolecular interactions, decreased hydrodynamic size or increased sample viscosity. There are reports that discuss the optimization of sample concentration and show that there is a critical concentration below which the effect of concentration is of little relevance and this critical concentration increases inversely with increasing MW [46,47]. Fortunately, concentration effects were minimized during the validation stage of the HPLC-SEC method in the concentration range of 100 to 1000 mg/L (according to correlation coefficients).

### 2.3. Molecular Distribution of HA Samples

**Figure 1:**
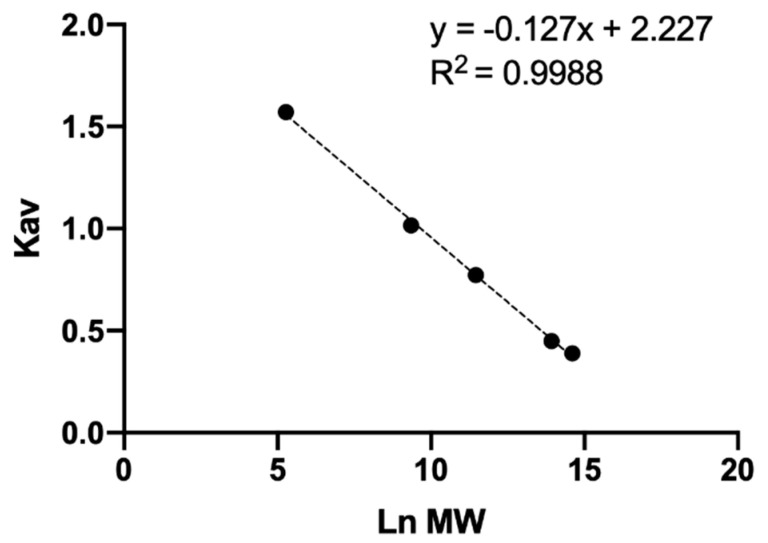
Analytical curve of molecular weight of HA.

Generally, when talking about a polymer, reference is made to a molecular weight distribution, this represents to count the real number of molecules that have the average molecular weight and how many have a higher or lower size, with this information molecular distribution curves are generated [48]. To evaluate the molecular distribution of unknown samples, the partition coefficient (Kav) of four standards of different average MW and D-glucuronic acid (monomer) was calculated. The Kav represents the fraction of stationary phase available for a given solute and is directly related to the size of the molecule and is independent of the column used so it is often used in analytical curves instead of the retention time [36,38,48]. To determine the Kav, it was necessary to calculate the exclusion volume (6.87 mL), total retention volume (13.75 mL), total column volume (14.34 mL), and void retention time (6.00 min) of the Ultrahydrogel 2000 column [36,38,48]. An analytical curve was developed to correlate Kav with the natural logarithm (Ln) of average MW (Figure 1). Multiple and adjusted coefficients of determination of 0.9988 and 0.9984 respectively showed a linear dependence among Kav and Ln MW. This analytical curve allowed the calculation of the average MW of the unknown samples.

**Figure 2:**
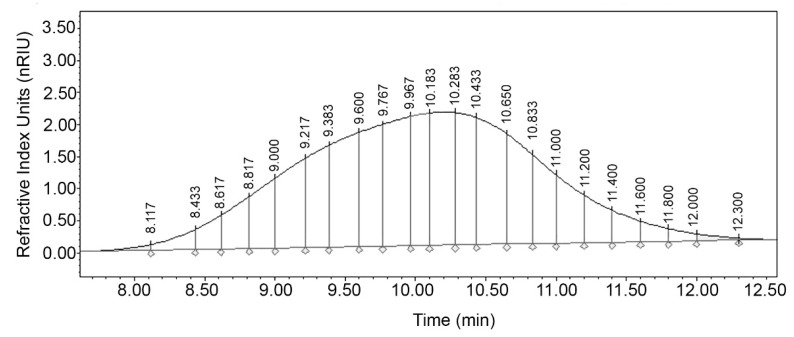
Integrated chromatogram with differential molecular weight distribution of HA sample at optimal conditions.

For the analysis of the molecular distribution, the differential areas associated with the degree of polymerization (DP) were calculated. The DPs represent the theoretical MW of a specific mass unit calculated according to the number of repeats of the HA dimer (D-glucuronic acid and N-acetyl glucosamine). With this information, the theoretical retention time ($t_{R,i}$) corresponding to each DP was calculated and the chromatographic peak of the sample HA was theoretically divided [48] in an exclusion range from 1 to 10,000 DP as seen in Figure 2. An increase in peak resolution is seen as the DP decreases mainly in fractions with a DP less than 100. Jagannath and Ramachandran [33] used an Ultrahydrogel 2000 column with average MW standards from 0.68 to 1.8 MDa and generated an analytical curve with an exponential fit. However, all samples with MW greater than 1.8 MDa were estimated by extrapolation, so it was not possible to calculate the polydispersity of the samples. In addition, there is no certainty that the data fit beyond the analyzed limit. In this study, we overcame these limitations during the validation stage of the analytical method, and it was possible to calculate important parameters in polymer analysis such as the number average molecular weight (${\bar{M}}_{n}$), weight average molecular weight (${\bar{M}}_{w}$), polydispersity (D), number $\left(\bar{D}P_{n}\right)$, and weight $\left(\bar{D}P_{w}\right)$ average degrees of polymerization.

### 2.4. HA Recovery by HPLC-SEC

The validation of the analytical method allows the evaluation of the concentration and molecular distribution of HA. However, some analytical methods often require prior extraction or purification steps of the analyte of interest and sometimes the recovery is not complete. Therefore, a sample of synthetic culture medium containing 200 mg/L HA (1.125 MDa) was analyzed to evaluate the recovery of HA, the results are shown in Table 3. For method 1 (M1), HA was extracted, precipitated with ethanol [49] and subsequently quantified by a photometric method [8]. With method 2 (M2), the HA was extracted and precipitated with ethanol (as described for method 1) and evaluated by the HPLC-SEC system. For method 3 (M3), the sample was only diluted and analyzed by the HPLC-SEC system. As expected, M3 allowed the best recovery of HA (99.11%) since the sample was not subjected to precipitation steps compared with the other methods, which represents a significant saving in analysis time and cost of organic solvents. In addition, it allows estimating the concentration and average MW simultaneously. M2 showed that ethanol precipitation did not achieve total recovery of the HA present in the sample with a loss of 7.06%. It has been shown that HA precipitation with organic solvents (usually ethanol or isopropanol) is a phenomenon dependent on pH, type and concentration of salt used for redissolution, solvent/culture broth ratio and viscosity [50]. Sousa, Guimarães, Gonçalves, Silva, Cavalcante and Azevedo [15] purified a sample of HA from bacterial culture broth with four cycles of precipitation/redissolution with ethanol/sodium nitrate to remove residual proteins and reported a loss in each of the precipitation steps with a total loss of 16% of the initial HA. This means that there is a loss of HA during the extraction and purification stages.

On the other hand, with M1, an overestimation of 12.84% was obtained for HA recovery that was attributed to impurities precipitated during the ethanol treatment, which means that it does not meet the international precision criteria required for any analytical method [34]. Traditional photometric methods do not analyze the complete HA polymer, require previous stages of purification with solvents, hydrolysis (acid, alkaline or enzymatic) and only quantify some of the HA monomers (D-glucuronic acid in this study) [8,10,12]. In addition, they do not evaluate MW and are sensitive to interferences by residual carbohydrates and proteins that produce false positives.

### 2.5. Concentration and Molecular Distribution of HA Samples by HPLC-SEC

**Figure 3:**
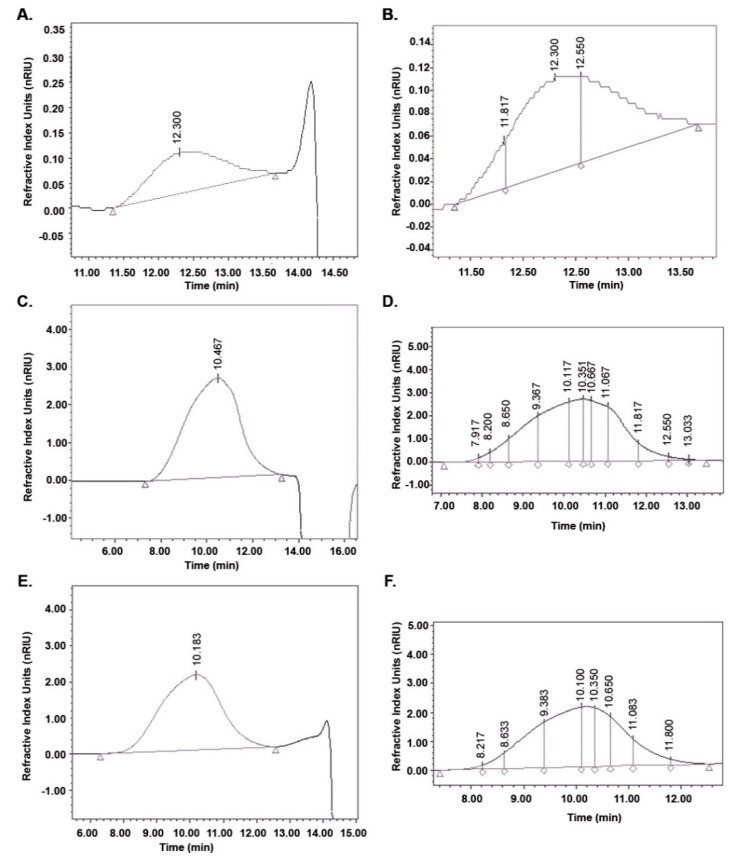
Chromatograms of HA in commercial samples and culture broth analyzed by HPLC-SEC. Chromatograms for serum, **A**; capsule, **C** and culture broth, **E**. Differential molecular weight distribution calculated for serum, **B**; capsule, **D** and culture broth, **F**.

**Figure 4:**
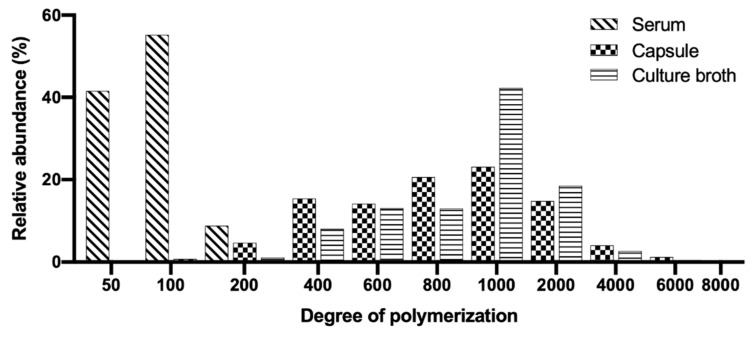
Histograms of HA in commercial samples and culture broth analyzed. Differential molecular weight distribution: molar fractions normalized to area = 100%.

To demonstrate the adaptability and robustness of the validated method HPLC-SEC, HA samples (cosmetic, pharmaceutical and culture broth) were analyzed. The parameters calculated are listed in Table 4 and the chromatograms are presented in Figure 3. The differential molecular weight distribution was also calculated in the chromatograms of each sample as shown in Figure 3. The HA present in the serum was the smallest in size (MW) and this agrees with that published by Alcalde and Del Pozo [51] for cosmetic products with an MW between 15,000 and 50,000 Da. The HA for cosmetic applications must be small so that the molecules can penetrate the skin to the epidermis [1]. The serum also presented the lowest polydispersity, whose molecular distribution is illustrated as a histogram in Figure 4 with DP values from 50 to 200.

For the capsule, a concentration of HA equivalent to that reported by the manufacturer (150 mg/capsule) and purity >98% was calculated. The capsule presented the highest molecular distribution of the samples (50 to 8,000 DP) and the values of ${\bar{M}}_{n}$ and ${\bar{M}}_{w}$ agree with those reported by Adam and Ghosh [52] for pharmaceutical products with HA of different origin (bacterial culture and rooster comb). The HPLC-SEC method allowed obtaining more resolved chromatographic peaks for HA from pharmaceuticals than HPLC-UV [22,24] and HPLC-MS [21]. The HA concentration in the culture broth was similar to the one reported by Jagannath and Ramachandran [33]. While in order to determine the MW, they extrapolated all the samples with a molecular distribution greater than 1.8 MDa so its determination could be inaccurate. The molecular distribution of HA from the culture broth was found to be between 200 and 6000 DP but the molecular fraction with the highest relative abundance (46%) corresponds to a theoretical molecular weight of 847,000 Da. This explains why ${\bar{M}}_{w}$ and $\bar{D}P_{w}$.are considerably higher than ${\bar{M}}_{n}$ and $\bar{D}P_{n}$.

## 3. Materials and Methods

### 3.1. Materials

Hyaluronic acid sodium salt (HA) from *Streptococcus equi* with an average molecular weight (MW) of 0.011, 0.095, 1.125 and 2.2 MDa, D-glucuronic acid, sodium dodecyl sulfate (SDS) and ethanol HPLC grade was purchased from Sigma Aldrich (St. Louis, MO, USA). Sodium nitrate analytical grade was kindly supplied by Fermont (Monterrey, NL, México). *Streptococcus equi* subsp. *zooepidemicus* (*S. zooepidemicus* #35246) were purchased from American Type Culture Collection (ATCC, Manassas, VA, USA). HA in culture broth was produced as reported by Chen, Chen, Huang and Chen [49]. HA in capsules (Women’s Hyaluronic acid, GNC, USA) and facial serum (Sérum Minéral 89, Vichy Laboratories, France) were purchased in a local pharmacy. According to the manufacturer, the qualitative composition of the capsules includes hyaluronic acid, gelatin, microcrystalline cellulose, and silica and the facial serum contains water, PEG/PPG/Polybutylene glycol-8/5/3 glycerin, glycerin, butylene glycol, methyl gluceth-20, carbomer, sodium hyaluronate, phenoxyethanol, caprylyl glycol, citric acid, and biosaccharide gum-1. Ultrapure water was obtained from a Millipore Milli-Q purification system (Burlington, MA, USA).

### 3.2. Experimental Setup for HPLC-SEC Analysis

The HPLC-SEC system included an Alliance e2695 module, Refractive Index (RI) 2414 detector, Empower 3 software and Ultrahydrogel 2000 column (7.8 × 300 mm) Waters (Milford, MA, USA), 0.1 M sodium nitrate mobile phase at a flow rate of 0.8 mL/min and 80 µL was injected for analysis. The method used was adapted from Jagannath and Ramachandran [33].

#### Column Temperature

To evaluate the effect of column temperature on the resolution of the peaks of interest, a sample of culture broth with HA was analyzed at four temperatures (30, 50, 60, 70 °C) and the linear correlation coefficient, repeatability and reproducibility were calculated according to the USP [34].

### 3.3. Evaluation of the Analytical Conditions of the HPLC-SEC Method

A 1 g/L stock solution of each of the HA and D-glucuronic acid standards was prepared by dissolving 5 mg in 5 mL of ultrapure water and stored at 4 °C. To validate the analytical method, seven concentration levels of the standards (100 to 1000 mg/L) were prepared with ultrapure water from the stock solution, filtered through 0.45 µm polyvinylidene fluoride (PVDF) membrane (Burlington, MA, USA) and analyzed on the HPLC-SEC system at 70 °C. HA concentration and peak area were plotted. Pearson’s correlation coefficient was calculated to estimate the fit of the experimental data to the analytical curve and statistical analysis was performed with Student’s *t*-test. The analytical method was evaluated by calculating repeatability, reproducibility, and limits of detection (LOD) and limits of quantification (LOQ) [34].

### 3.4. Molecular Distribution Analysis by HPLC-SEC

Number average molecular weight (${\bar{M}}_{n}$) and Weight average molecular weight (${\bar{M}}_{w}$) were determined. ${\bar{M}}_{n}$ is associated with the molar concentrations, whereas ${\bar{M}}_{w}$ is associated with the mass concentration. If all polymer chains were of equal length, ${\bar{M}}_{n}$ would be equal to ${\bar{M}}_{w}$ and the polymer would be monodispersed. The ratio between the values of ${\bar{M}}_{w}$/${\bar{M}}_{n}$ is known as polydispersity (D) and is used to know the heterogeneity of the polymer. The higher the value of D, the higher the molecular distribution of the sample [36,53,54]. To evaluate the molecular distribution, an analytical curve correlating the partition coefficient (Kav) and the natural logarithm of each HA standard was performed in a range from 0.011 to 2.2 MDa (600 mg/L concentration). The Kav was calculated with the Equation (1):

(1) $$Kav=\frac{V_{e}-V_{0}}{V_{T}-V_{0}}\;\;$$

where $V_{e}$ is the elution volume of the analyte, $V_{0}$ and $V_{T}$ are the void volume and total retention volume of the column respectively [38]. These parameters were calculated according to Huber and Praznik [36] and the molecular distribution was calculated according to Moreno-Vilet, Bostyn, Flores-Montano and Camacho-Ruiz [48], as described in Equations (2)–(6):

Number average molecular weight:

(2) $${\bar{M}}_{n}=\frac{\sum n_{i}M_{i}}{\sum n_{i}}$$

Weight average molecular weight:

(3) $${\bar{M}}_{w}=\frac{\sum n_{i}M_{i}^{2}}{\sum n_{i}M_{i}}$$

where n is the differential area under the curve in the chromatogram corresponding to the degree of polymerization (DP) and M is the theoretical molecular weight of the fraction i.

Polydispersity:

(4) $$D=\frac{M_{w}}{M_{n}}$$

Number average degree of polymerization of hyaluronic acid:

(5) $$DP_{n}=\frac{M_{n}-18}{846.8}$$

Weight average degree of polymerization of hyaluronic acid:

(6) $$DP_{n}=\;\frac{M_{w}-18}{846.8}$$

where 846.8 is the molecular weight of the HA dimer (D-glucuronic acid and N-acetyl glucosamine).

To determine the fraction i corresponding to the DP of HA with a specific molecular weight in the chromatogram, the theoretical retention time ($t_{R,i}$) was calculated with the Equation (7) according to Moreno-Vilet, Bostyn, Flores-Montano and Camacho-Ruiz [48]:

(7) $$t_{R,i}\;=\;K_{avtheo}\left(t_{T}-\;t_{0}\right)+\;t_{0}$$

where $t_{0}$ and $t_{T}$ are retention time of the void and total volume of column respectively. $K_{avtheo}$ is the theoretical retention coefficient calculated from the analytical curve parameters (slope and intercept).

### 3.5. HA Recovery

To evaluate HA recovery, synthetic culture medium was prepared according to Chen, Chen, Huang and Chen [49] and 200 mg/L of HA standard (1.125 MDa) was added. Three different methods were tested to quantify HA. For method 1 (M1), HA extraction was performed with 1 mL of sample and 1 mL of 0.1% (*w*/*v*) SDS, vortexed and allowed to react for 10 min at room temperature. Bacteria were removed by centrifugation at 10,000 rpm, 10 min (when bacterial culture samples were analyzed) and to 1 mL of supernatant was added 4 mL of HPLC-grade ethanol at 4 °C for 1 h. The HA was recovered by centrifugation at 8000 rpm 10 min, the precipitate was dissolved in distilled water and analyzed by the carbazole method [8]. With method 2 (M2) HA extraction was performed with 1 mL of sample and 1 mL of SDS, the bacteria were removed by centrifugation (when bacterial culture samples were analyzed) and the HA was precipitated with ethanol as described for method 1. The precipitated HA was dissolved in ultrapure water, filtered with 0.45 µm membrane and analyzed in the HPLC-SEC system. For method 3 (M3), 1 mL of sample was mixed with 1 mL of SDS, allowed to react and bacteria were removed under the conditions described above. The supernatant was filtered with 0.45 µm membrane and analyzed on the HPLC-SEC system. For each method, the concentration, average MW, and recovery rate of HA were calculated.

### 3.6. Analysis of Samples with HA

The concentration and molecular distribution of HA in culture samples and commercial products were determined. The culture broth sample was processed as described for M3. The contents of one capsule and one serum sample were dissolved with ultrapure water. All samples were filtered with 0.45 µm membrane and analyzed on the HPLC-SEC system.

## 4. Conclusions

In this study, an analytical method with a high degree of accuracy, was validated to simultaneously estimate the concentration and molecular distribution of HA by HPLC-SEC. It was demonstrated that, at 70 °C, the repeatability and reproducibility of HA quantification increased. The exclusion range of the analytical method is from 1 to 8000 degrees of polymerization (828 Da to 6.77 MDa), which includes HA obtained from different origins (animal tissue, bacterial culture, etc.) and commercial products (cosmetics, pharmaceuticals, etc.). The proposed analytical method represents a fast and accurate alternative to traditional methods involving solvent precipitation and hydrolysis processes of HA and can be applied in the determination of purity and molecular distribution of HA obtained from different origins, in the monitoring of production processes, and purification steps, for quality control, and evaluation of commercial products.

## Figures and Tables

**Table 1:** Repeatability and reproducibility of the HPLC-SEC method at different temperatures.

Temperature (°C)	Repeatability (%)	Reproducibility (%)
30	81.32	82.94
50	88.21	89.21
60	92.23	92.23
70	97.33	97.13

**Table 2:** Quantitative parameters for analytical validation of the HPLC-SEC method.

Parameter	Standard Average Molecular Weight (MDa)			
	0.011	0.095	1.125	2.200
Concentration (mg/L)	0–1000	0–1000	0–1000	0–1000
Repeatability (%)	98.35	98.22	99.01	99.01
Reproducibility (%)	98.37	98.29	99.05	99.20
LOD ^1^ (mg/L)	12.63	13.02	16.94	22.09
LOQ ^2^ (mg/L)	42.10	43.41	56.48	73.63
Analytical curve ^3^	y = 186.46x + 2191.2	y = 194.92x – 2745.2	y = 198.94x + 576.48	y = 224.27x + 766.48
PCC ^4^	0.99	0.99	0.99	0.99

^1^ Limit of detection; ^2^ Limit of quantification; ^3^ y = chromatographic peak area of the sample, x = mg/L of hyaluronic acid of the sample; ^4^ Pearson correlation coefficient.

**Table 3:** Recovery of HA with the different methods proposed.

Analysis Method	HA Recovery (mg/L)	Recovery Rate (%)	Average MW (Da)
M1	225.68 ± 11.16	112.84	NE ^1^
M2	185.88 ± 1.83	92.94	1,109,217.4
M3	198.23 ± 2.79	99.11	1,119,383.1

^1^ The method does not evaluate.

**Table 4:** Concentration and molecular weight distribution of HA in commercial samples and culture broth analyzed by HPLC-SEC.

Parameter	Sample		
	Serum	Capsule	Culture broth
HA content	1.2 ± 0.78 mg/100 mg	148.7 ± 3.1 mg/capsule	1351.6 ± 8.4 mg/L
Average MW (Da)	83,351.5 ± 813.3	683,662.9 ± 15,466.5	937,667.1 ± 10,174.1
${\bar{M}}_{n}$ ^1^ (Da)	77,372.1 ± 2773.6	912,577.4 ± 43,465.7	980,633.7 ± 7094.7
${\bar{M}}_{w}$ ^2^ (Da)	90,938.5 ± 470.0	1,481,244.2 ± 432,999.4	1,427,015.0 ± 8766.1
Polydispersity	1.18 ± 0.04	1.61 ± 0.4	1.46 ± 0.01
$\bar{D}P_{n}$ ^3^	91.3 ± 3.3	1077.7 ± 51.3	1158.0 ±8.4
$\bar{D}P_{w}$ ^4^	107.8 ± 0.6	1749.2 ± 511.3	1685.2 ± 10.4

^1^ Number average molecular weight; ^2^ Weight average molecular weight; ^3^ Number average degree of polymerization of HA; ^4^ Weight average degree of polymerization of HA.

## Data Availability

The data presented in this study are available upon request from the corresponding author (G.M.G.-M.).
